# A Dual-UNet Diffusion Framework for Personalized Panoramic Generation

**DOI:** 10.3390/jimaging12010040

**Published:** 2026-01-11

**Authors:** Jing Shen, Leigang Huo, Chunlei Huo, Shiming Xiang

**Affiliations:** 1 School of Artificial Intelligence, University of Chinese Academy of Sciences, Beijing 100049, China; 2 State Key Laboratory of Multimodal Artificial Intelligence Systems, Institute of Automation, Chinese Academy of Sciences, Beijing 100190, China; 3 Guangxi Key Lab of Human-Machine Interaction and Intelligent Decision, Nanning Normal University, Nanning 530100, China; 4 School of Information Engineering, Capital Normal University, Beijing 100048, China

**Keywords:** customized multi-view generation, panoramic synthesis, diffusion models, deep learning

## Abstract

While text-to-image and customized generation methods demonstrate strong capabilities in single-image generation, they fall short in supporting immersive applications that require coherent 360° panoramas. Conversely, existing panorama generation models lack customization capabilities. In panoramic scenes, reference objects often appear as minor background elements and may be multiple in number, while reference images across different views exhibit weak correlations. To address these challenges, we propose a diffusion-based framework for customized multi-view image generation. Our approach introduces a decoupled feature injection mechanism within a dual-UNet architecture to handle weakly correlated reference images, effectively integrating spatial information by concurrently feeding both reference images and noise into the denoising branch. A hybrid attention mechanism enables deep fusion of reference features and multi-view representations. Furthermore, a data augmentation strategy facilitates viewpoint-adaptive pose adjustments, and panoramic coordinates are employed to guide multi-view attention. The experimental results demonstrate our model’s effectiveness in generating coherent, high-quality customized multi-view images.

## 1. Introduction

In recent years, text-to-image generation models [1,2,3,4,5] have made significant breakthroughs, greatly advancing the field of image generation. Subsequently, customized image generation methods [6,7,8,9,10] such as DreamBooth [11] and Custom Diffusion [12] have emerged, allowing users to generate images based on specific references, aiming to meet customized requirements while preserving key features from the references. However, these studies primarily focus on single-image generation. In contrast, immersive applications such as virtual reality [13,14] and digital twins rely on coherent spatial perception, which often requires generating continuous multi-view images to construct a 360° panoramic environment. Methods like MVDiffusion [15] and Panfusion [16] can generate panoramas [17,18,19,20,21] or multi-view images from text, but they cannot incorporate user-specified images for customization. Although MV-Adapter [22] can generate multi-view images of a given object, it is essentially an object-level multi-view reconstruction model [23,24,25,26] and struggles to integrate objects naturally into complex panoramic scenes.

**Figure 1:**
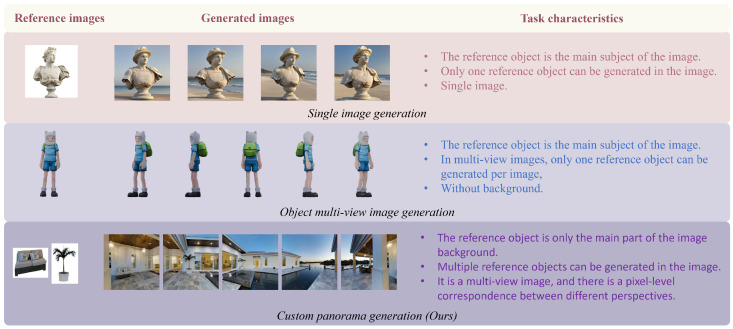
Differences between various tasks.

Furthermore, a common characteristic of both single-image customization and multi-view object customization is that the reference object occupies the dominant part of the image, and only one reference object can be generated, with little or no background interference. And in object-level multi-view generation, the correlations between images from different views are very high, allowing them to share features from the same reference image. In contrast, panoramas typically depict a scene where there is no dominant object; almost all elements appear as background components, and different views may contain different reference objects. This results in weaker correlations between images. Additionally, if a reference object appears in overlapping views, consistency across different perspectives must be maintained. These factors collectively increase the difficulty of customized panorama generation. The differences between these generation tasks are summarized in Figure 1.

Moreover, datasets for customized panorama generation are relatively scarce. First, compared to single images, panoramic image datasets for scene generation are inherently limited. Second, training customized generation models usually requires multiple paired images, which is even more challenging to obtain for panoramic data.

We propose a customized panorama image generation model, introducing a decoupled feature injection mechanism within a dual-UNet architecture to handle weakly correlated reference images across views, integrating spatial information by simultaneously feeding both reference images and noise into the denoising branch. A hybrid attention mechanism is used for the deep fusion of reference features and multi-view representations. Additionally, a data augmentation strategy is applied to enable viewpoint-adaptive pose adjustments. We also use panoramic coordinates to guide multi-view attention. The experimental results show the model’s effectiveness in generating high-quality, coherent personalized multi-view images.

The remainder of this paper is organized as follows: Section 2 introduces the related work. Section 3 describes the proposed method. The experimental results and analyses are presented in Section 4, followed by the conclusion in Section 5.

## 2. Ralated Work

### 2.1. Panorama Generation

The work on multi-view scene generation can be primarily categorized into two approaches: 2D-based methods and 3D-based methods. In the former, 2D images are directly generated by considering the 3D geometric constraints between different viewpoints. MVDiffusion [15] uses 8 parallel branches to generate images from 8 different viewpoints simultaneously and employs a Correspondence-Aware Attention (CAA) block to ensure viewpoint consistency. However, it requires predefined camera poses. CamFreeDiff [27] estimates the camera pose by predicting the homography transformation from the input view to a predefined canonical view, removing the need for predefined camera poses. PanoDiff [28] generates 360° panoramas from unregistered narrow field-of-view (NFoV) inputs. It estimates overlap between input pairs, projects them onto a panoramic canvas, and uses a latent diffusion model (LDM) to complete the panorama. AOGNet [29] introduces an autoregressive framework for 360° image generation using progressive outpainting with NFoV images and text guidance, but it lacks fine-grained control. PanFusion [16] uses two parallel branches to process panoramic and perspective images separately, and employs the Equirectangular Projection Perception (EPP) module to enable the model to capture correspondences between panoramic and perspective views. 3D-based methods reconstruct the scene in 3D space and generate multi-view images through rendering. Sat2Scene [18] integrates point clouds with a 3D diffusion model in three steps: extracting the scene’s geometry, applying sparse convolutions to color the foreground point cloud, generating the background with a 2D model, and using neural rendering to create high-quality images from arbitrary viewpoints.

### 2.2. Customized Image Generation

Currently, research on customized generation mainly focuses on two levels: single-image customization [11,30,31,32] and object-level multi-view [22] customization. These methods either generate customized images without maintaining multi-view consistency, or produce view-consistent images without customization. DreamBooth [11] customizes text-to-image diffusion models by fine-tuning them on a few images of a subject. It learns to associate a unique identifier with the subject, enabling the generation of realistic and diverse images of that subject in new contexts. IP-Adapter [31] introduces image prompting through a decoupled cross-attention [33] mechanism that independently attends to text and image features, allowing for more flexible integration of visual prompts. Paint by Example [30] incorporates a reference image by encoding it with CLIP [34] and injecting the features into the diffusion model using cross-attention, with spatial control guided by a mask. AnyDoor [32] enables object placement at specified locations and shapes within an image. It uses high-pass filters to capture the object’s high-frequency details and places them in the target region, followed by a ControlNet-style [35] UNet encoder to refine and enhance object appearance. MV-Adapter [22] is an object-level customized multi-view image generation model. It is a plug-and-play adapter that employs a decoupled attention mechanism, learning inter-view consistency through a parallel architecture combining reference image cross-attention and multi-view attention.

## 3. Method

### 3.1. Pipeline

The input of our model includes reference images, noise, panoramic coordinates, and multimodal prompts, where multimodal prompts are achieved by embedding the CLIP [34] features of the images into the text. The output of the model is continuous images from different viewpoints. The workflow begins by providing one or more reference objects and their positions in the images. These reference objects are then pasted into the corresponding positions to generate reference images that come from different viewpoints or different objects. Next, these reference images are encoded using a Variational Autoencoder [36]. The encoded images are then fed into a dual UNet architecture with a decoupled feature injection mechanism, which consists of a denoising branch and a reference branch. The denoising branch takes the encoded reference images concatenated with noise as input to enhance image quality and provide spatial guidance. The reference branch provides reference image features. The two branches process these features in parallel. Features extracted from both branches are integrated using a hybrid attention mechanism. Panoramic coordinate guidance and row-wise self-attention [37] is used to ensure viewpoint consistency.

The term “reference image” denotes the original target-only image in multimodal prompts, and otherwise represents a 512 × 512 composite image with the resized object positioned as specified, with this unified definition applied consistently throughout the paper.

### 3.2. Decoupled Feature Injection

**Figure 2:**
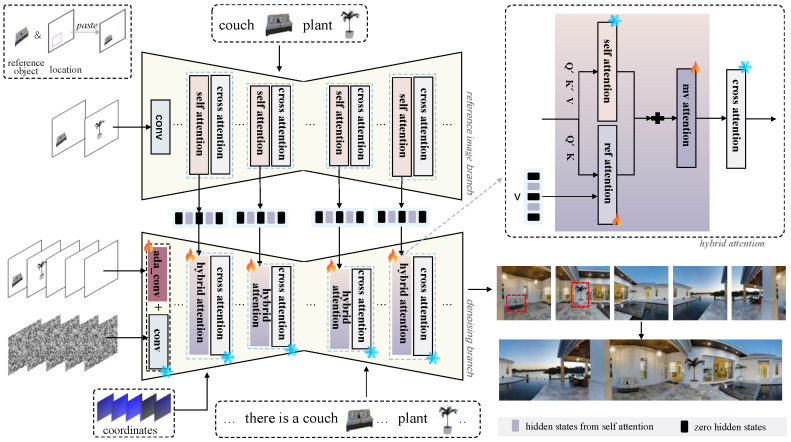
Pipeline. Reference images are fed into a dual-UNet architecture comprising denoising and reference branches. The denoising branch concatenates encoded images with noise to enhance quality and provide spatial guidance, while the reference branch extracts image features. Features from both branches are fused via a hybrid attention mechanism, with panoramic coordinate guidance ensuring viewpoint consistency.

In panoramic image generation, reference images from different viewpoints typically contain distinct objects with no inherent correlation. Therefore, unlike multi-view object generation, the same reference images cannot be used across different viewpoints. To tackle this issue, a decoupled feature injection mechanism is introduced within the dual-UNet architecture, aimed at managing weakly correlated reference images from different viewpoints, as shown in Figure 2. This mechanism processes reference images adaptively: when a reference is available for a target viewpoint, its features are extracted and injected into the denoising branch. Otherwise, zero-initialized feature vectors are employed to maintain network integrity. This conditional approach allows for flexible adaptation to references at arbitrary viewpoints, while ensuring stable generation in their absence. The feature injection is formally defined as follows:

(1) $$f_{\mathrm{ref}}=\left\{\begin{array}{cc} E(I_{v}), & \mathrm{if}\;\mathrm{reference}\;I_{v}\;\mathrm{exists}\\ 0, & \mathrm{otherwise} \end{array}\right.$$

where E denotes the self-attention layers in the reference branch. $f_{\mathrm{ref}}$ denotes the injected features for viewpoint *v*.

### 3.3. Hybrid Attention

**Figure 3:**
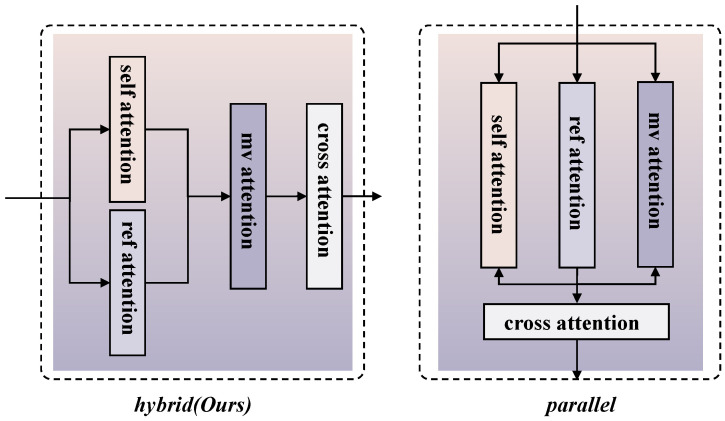
Hybrid (ours) vs. parallel.

MV-Adapter [22] employs a parallel structure to the original self-attention layer to fuse reference image features, with both its keys and values derived from reference image features and queries coming from noise branch features:

(2) $$f_{\mathrm{self}}=\mathrm{SelfAttn}\left(f_{\mathrm{in}}\right)+\mathrm{MVAttn}\left(f_{\mathrm{in}}\right)+\mathrm{RefAttn}\left(Q=f_{\mathrm{in}},\left(K,V\right)=f_{\mathrm{ref}}\right)+f_{\mathrm{in}}$$

where $f_{\mathrm{in}}$ is the input to the attention block, $f_{\mathrm{ref}}$ is the reference image features, SelfAttn is the original self-attention layer from the T2I model, MVAttn is the multi-view attention layer, and RefAttn is the reference image cross-attention layer, both created by duplicating the spatial self-attention layer. our model operates on multi-view images without explicitly injecting reference image features into every viewpoint. The parallel architecture of MV-Adapter suffers from progressive feature degradation, where visual cues present in the current view tend to deteriorate or disappear in adjacent generated views. To address this, we employ a hybrid attention mechanism that first performs deep fusion of reference image features and then enforces cross-view consistency constraints, as illustrated in Figure 3. Specifically, within the hybrid attention block, the reference image features are first added to the denoising features, and the combined features are then fed into the multi-view attention layer.

Furthermore, our denoising branch also takes reference image as input. The denoising branch is capable of computing attention scores by calculating similarities within itself, capturing the intensity of the reference image features that need to be obtained. Therefore, our keys and queries are derived from the noise branch features, and the values are derived from the reference image features. The aforementioned procedure can be mathematically formulated as follows:

(3) $$\begin{array}{c} f_{\mathrm{in}}=\mathrm{SelfAttn}\left(f_{\mathrm{in}}\right)+\mathrm{RefAttn}\left(\left(Q,K\right)=f_{\mathrm{in}},V=f_{\mathrm{ref}}\right) \end{array}$$

(4) $$\begin{array}{c} f_{\mathrm{self}}=\mathrm{MVAttn}\left(f_{\mathrm{in}}\right)+f_{\mathrm{in}} \end{array}$$

### 3.4. Multimodal Prompt Input

Our model incorporates reference images as input and employs a text-image multi-modal prompting mechanism that enables effective collaboration between prompt features and reference image features to facilitate target object generation. Textual information provides semantic guidance, while reference images supply visual cues. For the reference image branch, we use multi-modal prompts when reference images are provided, otherwise, an empty string. We encode images using CLIP and introduce a linear layer on image embeddings to facilitate modality alignment.

### 3.5. Data Augmentation

To enable our model to place objects at specified locations and adaptively adjust their pose, we faced the challenge that typical panoramic datasets lack images where only the object’s pose changes. To overcome this limitation, we employ a comprehensive data augmentation strategy on reference images to simulate diverse spatial configurations and improve model robustness. Each reference image is augmented with an 80% probability through a combination of resizing, padding, cropping, rotation, flipping, and geometric transformations, as detailed below.

- Resizing and padding: Each input image is resized to fit within a target resolution while preserving its aspect ratio, then padded with a white background to the exact target size.
- Random half cropping: With 20% probability, only half of the image—either the left or right side, chosen randomly—is retained. The cropped region is aligned to one side and vertically centered to simulate partial object views, thereby enhancing the model’s ability to complete occluded or missing regions.
- Random rotation: If not half cropped, the image is randomly rotated within $\pm 15^{\circ }$ with a 70% probability, using white padding to fill any empty regions introduced by rotation.
- Horizontal flip: Applied with 30% probability (if not half cropped), this operation mirrors the image horizontally to improve invariance to viewpoint direction.
- Geometric transformations: With 30% probability, a mild geometric distortion—either perspective or affine transformation—is applied, with deformation limited to 5% of the image dimensions to maintain structural consistency while increasing variability.

### 3.6. Panorama Coordinate Guidance

MV-Adapter relies on camera-to-object distance parameters, making it unsuitable for panorama generation due to significant depth variation among objects. Instead, we adopt equirectangular panoramic coordinates for implicit positional encoding. By computing the spherical coordinates of panorama pixels, we achieve a consistent scene representation without requiring explicit distance information.

For a panoramic image with resolution W×H, the spherical coordinates (θ,ϕ) of each pixel (u,v) are computed as follows:

(5) $$\begin{array}{c} \theta \left(u\right)=\frac{2\pi u}{W} \end{array}$$

(6) $$\begin{array}{c} \phi \left(v\right)=\frac{\pi v}{H}-\frac{\pi }{2} \end{array}$$

Here, θ∈[0,2π] denotes the azimuth angle, and ϕ∈[−π/2,π/2] represents the elevation angle. This mapping ensures continuity at the panorama boundaries by aligning θ=0 with θ=2π. To store the spherical coordinates as images, we normalize them into the [0,255] range:

(7) $$\begin{array}{c} \theta_{\mathrm{norm}}=\frac{\theta }{2\pi }\times 255 \end{array}$$

(8) $$\begin{array}{c} \phi_{\mathrm{norm}}=\frac{\phi +\pi /2}{\pi }\times 255 \end{array}$$

The normalized coordinate panorama is then converted into five perspective views, serving as implicit positional guidance for consistent multi-view generation. This positional guidance is added to the attention outputs of the UNet.

## 4. Experiments

### 4.1. Experimental Setup

We conduct experiments on the Matterport3D [38] dataset, which contains 10,912 indoor panoramas. Following the MVDiffusion data split, 9820 panoramas are used for training and 1092 for evaluation. Each panorama is projected with a field of view (FoV) of $90^{\circ }$ and a yaw step of $72^{\circ }$. Experiments are conducted under two configurations, with 5 and 15 viewpoints, respectively. In the 5-view setting, five central horizontal viewpoints are evenly distributed along the horizontal plane, corresponding to yaw angles $\theta =[0^{\circ },72^{\circ },144^{\circ },216^{\circ },288^{\circ }]$ and pitch angles $\phi =[0^{\circ },0^{\circ },0^{\circ },0^{\circ },0^{\circ }]$. In the 15-view setting, additional viewpoints are added above and below the central horizontal row to form three vertical levels (top, middle, and bottom), each containing five evenly spaced directions. The corresponding yaw and pitch angles are

$$\begin{array}{cc} \theta & =[0^{\circ },72^{\circ },144^{\circ },216^{\circ },288^{\circ },0^{\circ },72^{\circ },144^{\circ },216^{\circ },288^{\circ },0^{\circ },72^{\circ },144^{\circ },216^{\circ },288^{\circ }],\\ \phi & =[60^{\circ },60^{\circ },60^{\circ },60^{\circ },60^{\circ },0^{\circ },0^{\circ },0^{\circ },0^{\circ },0^{\circ },-60^{\circ },-60^{\circ },-60^{\circ },-60^{\circ },-60^{\circ }]. \end{array}$$

Captions are generated using BLIP-2 [39], and target objects are detected using YOLOv8n [40], with only high-confidence and size-qualified objects from selected categories retained. The 5-view model is trained on four NVIDIA RTX 3090 GPUs (24GB VRAM), and the 15-view model on four NVIDIA RTX 4090 GPUs (48GB VRAM).

We employ Fréchet Inception Distance (FID) [41], Inception Score (IS) [42], CLIP Score (CS) [34], and Peak Signal-to-Noise Ratio (PSNR) as our evaluation metrics. In Table 1, we also report GPU memory usage (Mem(G)) and inference time (Time(s)) for each method.

### 4.2. Training and Inference Details

We specifically filter for objects belonging to the following categories: chair, bed, couch, potted plant, tv, car, toilet, dining table, and sink, retaining only those instances with detection confidence scores above 0.82. To ensure high-quality reference images, we apply additional size constraints: each region of interest (ROI) must have a longer dimension exceeding 100 pixels and a shorter dimension greater than 50 pixels. The selected ROIs then undergo background removal, resulting in clean, object-focused reference images optimized for customized generation tasks.

**Figure 4:**
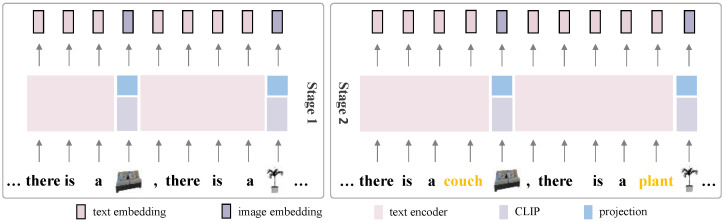
Multi-modal prompts at different training stages.

For both 5-view and 15-view models, we adopt stable-diffusion-2-1-base as the foundational generative model, with an image resolution of 512×512. Training is conducted using the AdamW optimizer with a learning rate of $1\times 10^{-4}$. A DDPM scheduler is employed for noise scheduling during both training and inference. Our model is trained using the standard L2 noise prediction loss, without any additional auxiliary losses We adopt a two-stage training strategy: the model is first trained on the 5-view setting for 50 epochs using prompts where the category name is replaced by the corresponding image embedding; then, the category text is added and the model is trained for 1 additional epoch, as illustrated in Figure 4. The 15-view model is initialized from the 5-view model and further trained. The 5-view model was trained using 4 24GB NVIDIA RTX 3090 GPUs (NVIDIA Corporation, Santa Clara, CA, USA), and the 15-view model was trained using 4 48GB NVIDIA RTX 4090 GPUs (NVIDIA Corporation, Santa Clara, CA, USA).

In both training and inference phases, if the reference image category name does not appear in the prompt, the reference image feature is not embedded into the textual prompt, and the reference image is only introduced through the dual-UNet architecture by feeding it into each branch separately.

### 4.3. Customized Multi-View Image Generation

Baseline. Our model has multi-view image generation capability, and we selected three related methods for comparison. A brief description is as follows:

Text2Light [19] is a zero-shot model for generating panoramas from text.

MVDiffusion [15] is a multi-view image generation model, but it generates eight views (FOV = 90°, ROT = 45°).

PanFusion [16] is an end-to-end model trained on Matterport3D [38] to generate panoramic images.

We conduct experiments under 5-view and 15-view settings, generating the corresponding perspective views from each model’s output for comparison. Column-wise attention is incorporated in the 15-view model to enforce vertical viewpoint consistency.

Results. Table 1 demonstrates that our model trained with 5 views surpasses all baseline methods across FID, IS, CS, and PSNR metrics. And our model achieves the fastest inference speed with low GPU memory consumption. Specifically, compared to PanFusion, our reference-based model achieves a relative improvement of 17.6% in FID, decreasing from 25.39 to 20.91, and consistently attains higher IS, CS, and PSNR, indicating improved visual quality and structural consistency. Notably, despite using fewer parameters and significantly lower GPU memory than PanFusion (6.5G vs. 15.4G), our method achieves superior performance with an inference time of only 10 s, demonstrating a favorable trade-off between efficiency and generation quality. In addition, even without reference images, our model maintains competitive performance across all metrics, suggesting that the proposed framework does not rely heavily on reference inputs and remains robust in reference-free scenarios.

Table 2 compares the model trained with 15 views under 15-, 5-, and 8-view configurations, showing superior or competitive performance across all configurations. Under the 15-view evaluation, our model achieves the best FID and CS among all methods, indicating its strong capability to model complex multi-view geometric relationships. When evaluated under the 5-view configuration, the 15-view trained model remains competitive and outperforms all baseline methods in FID and CS, demonstrating effective cross-view generalization. Interestingly, although the model is not trained on the 8-view setting, it still achieves the best FID and CS, highlighting its robustness to unseen view counts and flexible deployment under varying inference conditions. Furthermore, we report the results for two reference-free models, which outperform baseline methods in the 5-view setting and demonstrate strong performance in the 15-view configuration. Although our model was not trained on 8 views, it still obtains the best FID and CS, indicating strong generalization ability. A vertical comparison under the 5-view evaluation setting shows that the model trained on 15 views slightly underperforms the one trained specifically on 5 views. This may be due to the non-central views in the Matterport3D dataset containing more blurry or structurally simple content, which leads the model to learn less informative patterns.

**Figure 5:**
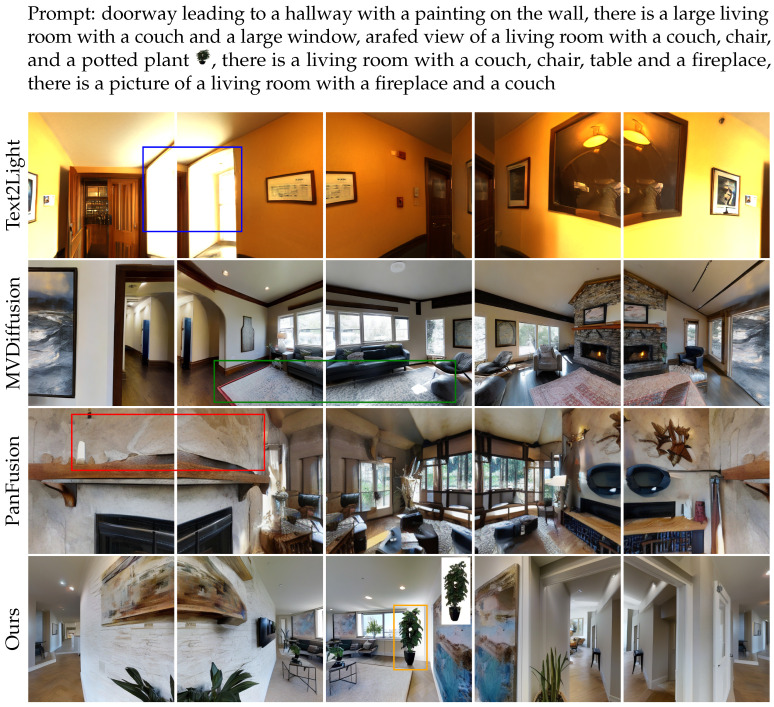
Five-view generation results of different methods, with problematic regions in baseline methods highlighted using colored boxes: inconsistent lighting, color mismatch, incomplete or blurry objects. Our method mitigates these issues and provides a customized multi-view image.

**Figure 6:**
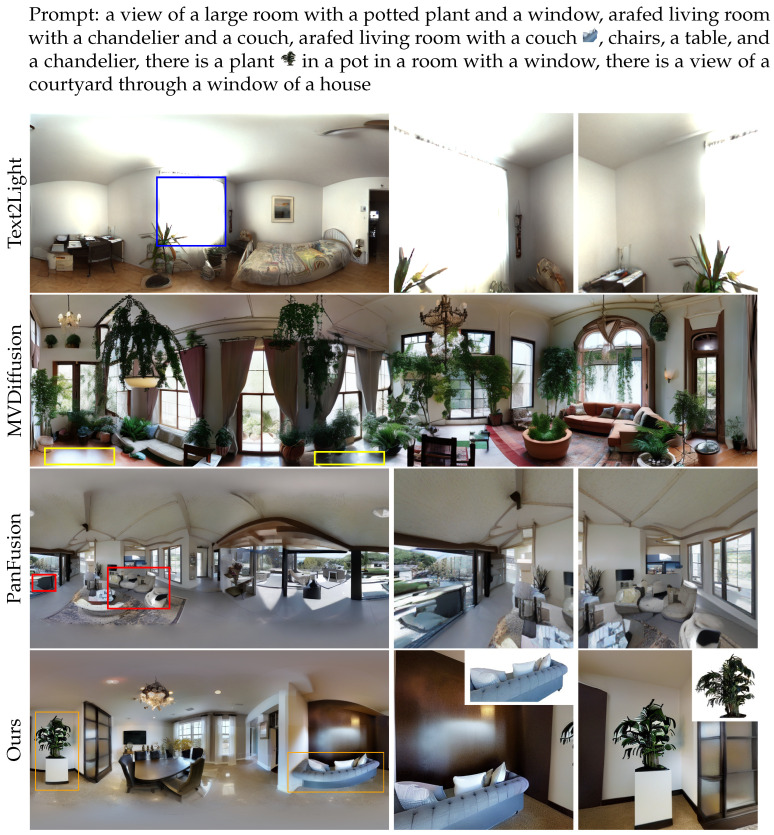
Fifteen-view generation results of different methods. The generated panorama highlights problematic regions in baseline methods with colored boxes: inconsistent lighting, color mismatch, and incomplete or blurry objects. Our method mitigates these issues and provides a customized multi-view image. Perspective projections for some issues are provided.

Figure 5 and Figure 6 present qualitative comparisons of the 5-view and 15-view models, along with the results from various baseline methods. In particular, the results of the 15-view model are presented using both full panoramas and zoomed-in perspective views from specific locations. Compared to Text2Light, our method produces more natural lighting and richer scene content. Compared to MVDiffusion, our model ensures better continuity of the same object across different viewpoints. Compared to PanFusion, our results exhibit higher realism and more complete object structures. Additionally, unlike previous methods that rely on a single reference image, our model is capable of incorporating multiple reference images, enhancing its flexibility and ability to generate more diverse customized views.

### 4.4. Ablation Study

Ablation experiments are conducted under the 5-view setting. Specifically, we compare two variants: one without the data augmentation mechanism, and another where the hybrid attention structure is replaced with a parallel attention design, evaluating the contributions of each component.

w/o Aug. In this experimental configuration, the reference image is simply resized and placed at a fixed location without any data augmentation. All other settings remain unchanged.

Parallel Attn. In this experimental configuration, only the hybrid attention is replaced with parallel attention, with data augmentation retained. All other settings are kept the same.

**Figure 7:**
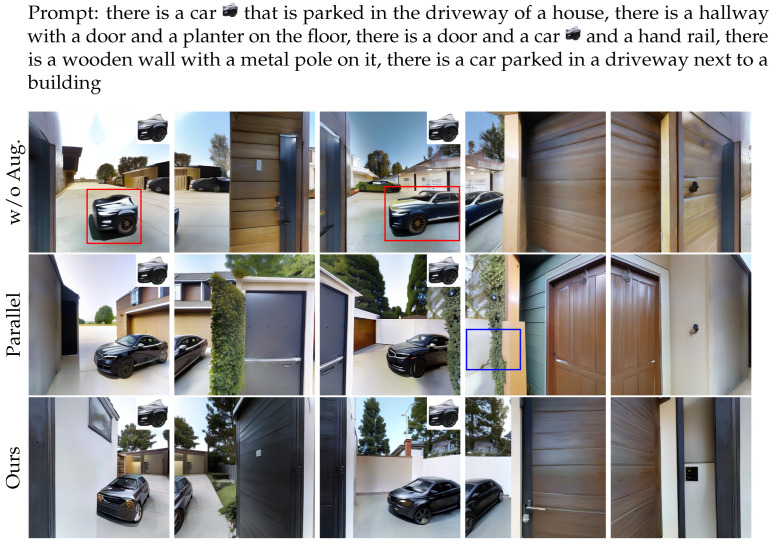
Ablation study results. We show the perspective views of 5 consecutive viewpoints from different settings, with problematic regions in baseline methods highlighted using colored boxes: incomplete or failed view-adaptive, feature degradation.

**Results.** Table 3 presents the results of the ablation study, demonstrating that the proposed components improve the overall performance. The visualization results in Figure 7 show that our hybrid attention module effectively merges reference image features with multi-view consistency constraints, ensuring continuous viewpoint alignment. Additionally, our data augmentation mechanism equips the model with viewpoint-adaptive capabilities.

**Hybrid attention.** Compared to the parallel architecture, our hybrid attention achieves better FID, IS, and PSNR. As shown in Figure 7, parallel architecture suffers from feature degradation during viewpoint transitions when reference images appear in overlapping view regions. This issue stems from the parallel attention architecture’s decoupled mechanism for reference feature injection and multi-view consistency constraints. Essentially, while the model enforces view consistency, it does not explicitly apply these constraints to the reference image features. As a result, the reference image features needed for adjacent views are obtained implicitly through the model’s multi-view consistency mechanisms, lacking persistent cross-view guidance. This leads to the degradation of key visual cues from the reference image during view transitions. A possible consequence of this behavior is that the generated images sometimes rely more heavily on the textual input rather than the reference images, which can explain why CS scores for the parallel architecture are occasionally higher than for our hybrid attention, despite lower performance on other metrics. In contrast, our hybrid attention mechanism first fuses the reference image features with the original features before applying multi-view constraints. This explicitly propagates the visual information from the reference image to other views, ensuring the persistent injection of reference features. As a result, it preserves detailed features from the reference image while enhancing inter-view coherence, leading to smoother viewpoint transitions and higher-quality image generation.

**Data augmentation.** The quantitative results demonstrate that the data-augmented model achieves comprehensive performance improvements across all four evaluation metrics compared to its non-augmented counterpart. The visualization results in Figure 7 show that the model without data augmentation simply copies the reference image and fails to adapt to viewpoint changes. In contrast, the model with data augmentation effectively adjusts object morphology to respond to perspective variations and can faithfully reconstruct complete objects from incomplete references.

**Multimodal prompts.** Regarding the overall effectiveness of the multimodal prompt, ablation experiments comparing different approaches are presented in Table 4. Overall, the model performs better when using the multimodal prompting strategy.

**Figure 8:**
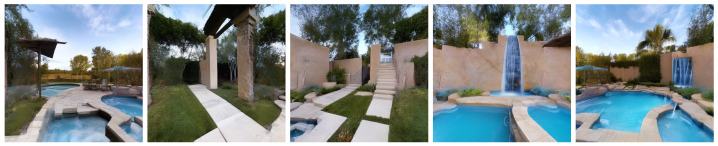
Ablation study on the effect of panorama coordinate guidance. Removing this guidance causes severe inconsistencies and spatial misalignment across viewpoints, demonstrating its crucial role in maintaining multi-view coherence.

**Panorama coordinate guidance.** We also conducted an ablation experiment by removing the panorama coordinate guidance. As shown in Table 5 and Figure 8, the absence of this guidance leads to severe inconsistencies across different viewpoints, significantly affecting spatial alignment. This validates the importance of panorama coordinate guidance in maintaining viewpoint consistency.

### 4.5. Further Analysis

**Figure 9:**
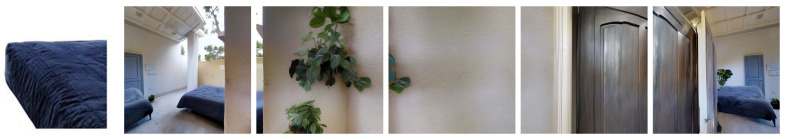
Visualization of occlusion cases.

Our model has the ability to handle occlusion. In Figure 9, given a partially occluded bed with the prompt “behind a wall”, the model accurately reconstructs the occluded scene, and can also generate a complete bed from only a partial input and a basic object prompt.

We also compared the quality of the generated reference images, as shown in Table 6. Our model effectively preserves the features of the reference image.

## 5. Conclusions

In this work, we explore the integration of reference images into multi-view image generation for indoor panoramic scenes, presenting a customized multi-view image generation model for such settings. Unlike previous object-level approaches, our model addresses challenges where the reference object may occupy only a small region of the image, and different views may require distinct references. In contrast to previous multi-view generation models, our method is capable of producing personalized images. Through the proposed decoupled reference feature injection across views, hybrid attention mechanism, and data augmentation strategy, our model achieves strong performance. Extensive experiments demonstrate that our method produces coherent, high-quality, and personalized multi-view images within indoor panoramic environments. While the design of our method is general, its extension to outdoor or non-architectural scenes would primarily depend on the availability of corresponding datasets.

Limitations. Due to dataset constraints, the model’s ability to adapt to varying object poses and viewpoints still requires improvement. Furthermore, our method relies on an object detector and a background removal pipeline to extract clean reference images, which constitutes another limitation.

## Figures and Tables

**Table 1:** Comparison with SoTA methods in 5-view setting. The up/down arrows (↑/↓) in the table are standard notation indicating the direction of better performance (higher/lower values, respectively).

Method	Custom	FID↓	IS↑	CS↑	PSNR↑	Mem(G)↓	Time(s)↓
Text2Light	✗	54.67	5.53	23.97	8.05	**3.9**	65
MVDiffusion	✗	–	–	–	–	7.9	46
PanFusion	✗	25.39	6.64	25.08	10.91	15.4	46
Ours (w/Ref.)	✓	**20.91**	**6.70**	**25.79**	**11.40**	6.5	**10**
Ours (w/o Ref.)	✓	**21.79**	**6.69**	**25.54**	**11.40**	6.5	**10**

(w/Ref.): with reference images; (w/o Ref.): without reference images. Bold indicates the best result.

**Table 2:** Comparison with SoTA methods in 15-view setting. The up/down arrows (↑/↓) in the table are standard notation indicating the direction of better performance (higher/lower values, respectively).

Method	15 Views			5 Views			8 Views		
	FID↓	IS↑	CS↑	FID↓	IS↑	CS↑	FID↓	IS↑	CS↑
Text2Light	53.50	6.45	23.78	54.75	5.48	23.93	51.84	5.73	23.76
MVDiffusion	–	–	–	–	–	–	25.27	**6.90**	26.34
PanFusion	24.84	**7.03**	24.64	26.21	6.63	24.21	23.84	6.72	24.22
Ours (w/Ref.)	**20.93**	6.92	**27.51**	**25.65**	**6.76**	**28.01**	**23.55**	6.16	**26.80**
Ours (w/o Ref.)	**21.50**	6.99	**27.53**	26.72	**6.99**	**27.99**	24.51	6.50	**26.72**

(w/Ref.): with reference images; (w/o Ref.): without reference images. Bold indicates the best result.

**Table 3:** Ablation study of model components under the 5-view setting. The up/down arrows (↑/↓) in the table are standard notation indicating the direction of better performance (higher/lower values, respectively).

Method	FID↓	IS↑	CS↑	PSNR↑
w/o Aug.	23.63	5.94	25.59	11.09
Parallel Attn.	21.47	6.34	**25.90**	10.82
Ours (w/Ref.)	**20.91**	**6.70**	25.79	**11.40**

(w/Ref.): with reference images. Bold indicates the best result.

**Table 4:** Ablation study of multimodal prompts under the 5-view setting. The up/down arrows (↑/↓) in the table are standard notation indicating the direction of better performance (higher/lower values, respectively).

Method	FID↓	IS↑	CS↑
removing the reference branch image prompt	23.66	7.01	25.67
removing the denoising branch image prompt	23.62	7.02	25.69
using pure text in both branches	23.67	**7.03**	25.69
Ours (w/Ref.)	**20.91**	6.70	**25.79**

(w/Ref.): with reference images. Bold indicates the best result.

**Table 5:** Ablation study of panorama coordinate guidance under the 5-view setting. The up/down arrows (↑/↓) in the table are standard notation indicating the direction of better performance (higher/lower values, respectively).

Method	FID↓	IS↑	CS↑
removing panorama coordinate guidance	22.61	5.97	25.56
Ours (w/Ref.)	**20.91**	**6.70**	**25.79**

(w/Ref.): with reference images. Bold indicates the best result.

**Table 6:** Performance of the reference image. The up arrows (↑) in the table are standard notation indicating the direction of better performance (higher).

Method	IS↑	CS↑
Reference images	5.70	**26.82**
Entire images	**6.70**	25.79

Bold indicates the best result.

## Data Availability

Restrictions apply to the availability of these data. Data were obtained from Matterport3D and are available at https://matterport.com/ (accessed on 25 August 2024) with the permission of Matterport3D.
